# Effect of Dietary Supplementation of *Moringa Oleifera* on the Production Performance and Fecal Methanogenic Community of Lactating Dairy Cows

**DOI:** 10.3390/ani9050262

**Published:** 2019-05-22

**Authors:** Lifeng Dong, Tingting Zhang, Qiyu Diao

**Affiliations:** Beijing Key Laboratory for Dairy Cow Nutrition, Sino-US Joint Lab on Nutrition and Metabolism of Ruminants, Feed Research Institute, Chinese Academy of Agricultural Sciences, Key Laboratory of Feed Biotechnology of the Ministry of Agriculture, Beijing 100081, China; donglifeng@caas.cn (L.D.); tingting5_2_0@163.com (T.Z.)

**Keywords:** *Moringa oleifera*, fecal methanogenic community, dairy cows, *mcr*A gene sequencing technique

## Abstract

**Simple Summary:**

High-quality forages such as protein-rich ingredients are essential to maximize production performance in dairy production. However, enteric methane produced by methanogenesis represents a substantial waste of feed energy for ruminants. Thus, it is important to evaluate the environmental effect when such feed ingredients are used to provide necessary nutrients. The aim of the present study was to examine the effects of dietary supplementation of *Moringa oleifera* on the production performance and fecal methanogenic community in lactating cows. The study’s main results suggest that inclusion of *Moringa oleifera* improved milk fat content and changed the composition and diversity of methanogens. This study indicates that secondary metabolites from *Moringa oleifera* may regulate fermentation conditions and associations between some methanogens and other microbes. These findings provide basic information on the utilization of alternative forage resources for dairy cows and can help to better understand the regulation of microbial metabolic function and methane emissions.

**Abstract:**

Development of alternative forage resources is of great importance to provide necessary nutrients and minimize greenhouse gas emissions in ruminant production. The aim of this study was to examine the effects of dietary supplementation of *Moringa oleifera* on the production performance and fecal methanogenic community in dairy cows using methyl-coenzyme M reductase α-subunit gene. Sixty-four cows were allocated to one of four treatments: basal diet without *M. oleifera* (control) or low (3% *w/w*, M3), medium (6%, M6), or high (9%, M9) supplementation with *M. oleifera*. This study demonstrated that different supplementation levels of *Moringa oleifera* in the diet achieved similar feed intake and milk production, but adding 6% of *Moringa oleifera* improved milk fat content. Two families, two phyla, three genera, and three species in total were identified among the four treatments. The fecal archaeal community in the control treatment was predominated by *Methanobrevibacter* (39.1% of the total sequence reads) followed by *Methanosphaera* and *Methanocorpusculum* at the genus level. The increased abundance of the *Methanosphaera* genus and *Methanosphaera* sp. ISO3-F5 species was induced by secondary metabolites of *Moringa oleifera* in the diet. Results indicated that *Moringa oleifera* supplementation not only improved dairy product quality but could also potentially reduce methane emissions.

## 1. Introduction

Forage source and nutrient composition hold significant importance for dairy production systems due to the constant cost of commercial concentrates. This cost is a serious constraint for smallholder farms when the dietary protein sources are restricted or the cost is unaffordable. In the past few decades, many efforts have been made to explore less-expensive ingredients such as agricultural by-products, tree foliage, and plant leaves to supply adequate nutrients [1]. 

As an indigenous native tree in the Himalayas, *Moringa oleifera* (*M. oleifera*) is a perennial leafy tree that produces a high biomass in a short period and is widely distributed in tropical and subtropical areas around the world [2]. Recently, it has been increasingly considered as an alternative ingredient for animal feed because of its high content of protein, vitamins, and minerals. The average crude protein (CP) contents of *M. oleifera* range from 180 to 270 g CP/kg DM, similar to that of sesame meal (260 g CP/kg DM) [1]. In addition, saponins, tannins, and polysaccharides in *M. oleifera* demonstrate beneficial anti-inflammatory, antioxidant, and antimicrobial activities and can increase milk yield when dairy cows are offered dried or fresh leaves and soft twigs [3,4]. A recent study [5] found that alfalfa hay and maize silage can be partially replaced by *M. oleifera* silage without negative effects on nutrient digestibility and milk yield. This enhanced production performance was due to the considerable amounts of secondary metabolites in *M. oleifera*, which were also used as a potential feed additive to reduce greenhouse gas (GHG) emission of ruminants.

Methane (CH_4_) emission from ruminants is a major contributor to atmospheric CH_4_ accumulation. Reduction of ruminal CH_4_ emissions would thus improve energy utilization efficiency and alleviate environmental issues within the dairy industry. For example, ruminal CH_4_ emissions were significantly decreased when *M. oleifera* leaves were used in an in vitro experiment [6]. Similarly, supplementation of pomegranate pulp in the diet decreased enteric CH_4_ emissions in dairy cows [7]. These reductions in CH_4_ emissions were attributed to the direct reduction of ruminal methanogenesis by active polyphenolic compounds either in *M. oleifera* leaves or pomegranate pulp. However, the variation and responses of fecal methanogenic community to *M. oleifera* supplementation in the diet of dairy cows have not been elucidated. 

Archaeal methanogens are obligate anaerobes that use methanogenesis pathways to facilitate fiber digestion by converting hydrogen into CH_4_. Daquiado et al. [8] found the predominant species in rumen fluid and manure was *Methanobrevibacter ruminantium* (63.6% and 62.4%, respectively), whereas *Methanocorpusculum labreanum* was most abundant in rectal dung for beef cattle (53.2%). In addition, the community structures of fecal microbiota reflect not only animal productivity but also health and food safety. Jin et al. [9] found that dietary supplementation of active dried yeast significantly increased the relative abundance of *Methanocorpuschulum* and *Thermoplasma* species but decreased *Methanobrevibacter* in the feces. Mohammadzadeh et al. [10] observed a decrease in the fecal methanogenic archaea abundance in goats when the diet changed from alfalfa hay to a combination of alfalfa hay and oats. Determining the fecal methanogen composition would help us understand the effect of dietary supplementation on methanogenesis and CH_4_ emissions. Therefore, the objective of the present study was to determine the effect of dietary supplementation of *M. oleifera* on the production performance and on the population and diversity of the fecal methanogenic community in lactating Holsteins dairy cows.

## 2. Materials and Methods 

This study was conducted at the Guoxiu dairy farm located in Boading, China (latitude: 38°45′54″ and longitude: 115°08′06″) in 2017. The experiment design and animal care and handling procedures were evaluated and approved by the Animal Ethics Committee of the Chinese Academy of Agricultural Sciences (protocol number 023-2017) prior to the commencement of the experiment.

### 2.1. Animals and Experimental Design

Sixty-four multiparous lactating Holstein dairy cows (120 ± 8.0 days in milk; 31.9 ± 1.20 kg/day of milk yield at the beginning of the trial) were used in this experiment. Animals were randomly assigned to one of four treatments: (1) control, basal diet without *M. oleifera*; (2) a low supplementation of *M. oleifera* (3% *w/w*; M3); (3) medium supplementation of *M. oleifera* (6% *w/w*; M6); (4) high supplementation of *M. oleifera* (9% *w/w*; M9). The basal diet was formulated to be isoenergetic and isonitrogenous to meet the nutrient requirements of lactating dairy cows (NY/T 34-2004, Table 1). The treatments were balanced for milk yield, body weight, and lactation period. Rachises and twigs of *M. oleifera* at 56 days of age were harvested in Guangdong, China (23°8′ N, 113°17′ E); these materials were then chopped and dried on plastic sheets for 3 days for further preparation of a total mixed ration (TMR) diet. The CP, neutral detergent fiber (NDF) and acid detergent fiber (ADF) content of *M. oleifera* were 71.5, 743 and 552 g/kg DM, respectively, while the ether extract (EE) and ash content was 48.6 and 78.3 552 g/kg DM, respectively. The TMR diet was offered ad libitum in amounts resulting in 5% refusals. The whole experiment (77 days) consisted of 14 days for adaptation to the diet and 63 days for feeding period, with fecal samples collected in the last 5 days of the feeding period. Animals were fed with TMR at 07:00 and 19:00 and milked twice at 06:00 and 16:00 on a daily basis. Cows were housed in individual tie-stalls in a barn with good ventilation and had continuous access to water throughout the experiment. Artificial light was provided by suspended bulbs, and the floor was cleaned twice daily for good hygiene.

### 2.2. Sample Collection and Measurements 

Samples of *M. oleifera*, TMR, and refusals were collected daily. All samples were composited and analyzed for DM (65 °C in a forced-air oven to a constant weight), CP (method 990.03; AOAC International, 2016), ADF and NDF (Ankom200 Fiber Analyzer, Ankom Technology, Macedon, NY, USA). Ash and EE concentration was determined with method 942.05 (AOAC International, 2016) [11]. Milk samples were recorded in the last 5 days of the feeding period and treated with 2-bromo-2-nitropropane-1-2-diol for determination of milk protein, fat, lactose, and total solids. Energy-corrected milk yield (ECM), standardized to 4.0% fat and 3.3% protein, was calculated using the equation below:

(1) $$\mathrm{ECM}\;\left(\mathrm{kg}/\mathrm{cow}\;\mathrm{per}\;\mathrm{day}\right)=mikl\;yield\;\left(\mathrm{kg}/d\right)\times \frac{376\times fat\;\%+209\times protein\;\%+948}{3138}.$$

Rectal fecal samples (200 g per sampling) were collected four times a day (08:00, 12:00, 16:00, and 20:00) and bulked per animal according to institutional animal care guidelines. Fecal samples were stored at −20 °C for later DNA extraction, high-throughput sequencing, and bioinformatics analysis.

### 2.3. DNA Extraction and High-Throughput Sequencing using McrA Gene

Fecal samples were freeze-dried, and total DNA was extracted from 200 mg samples with a QIAamp Fast DNA Stool Mini Kit (QIAGEN, CA, USA) according to the manufacturer’s instructions. DNA extracts were dissolved in 200 μL elution buffer and the quality and quantity of the extracted DNA were determined using a NanoDrop ND-1000 Spectrophotometer (Nyxor Biotech, Paris, France). The PCR primers used to amplify the *mcr*A fragments were from those of Luton et al. [12]: 5′-GGTGGTGTMGGATTCACACARTAYGCWACAGC-3′ (forward) and 5′-TTCATTGCRTAGTTWGGRTAGTT-3′ (reverse). The PCR was performed using the TaKaRa rTaq DNA Polymerase system and 2 μL of 10× buffer, 2 μL of deoxynucleotide triphosphate (dNTPs) mixture (2.5 mmol/L), 0.2 μL of rTaq polymerase, and 0.8 μL of each primer (forward and reverse). This reaction mixture (25 μL) used the following program: 95 °C for 3 min, followed by 30 cycles of 95 °C for 30 s, 55 °C for 30 s, and 72 °C for 45 s, and a final extension of 72 °C for 10 min. PCR products were electrophoresed in 1% agarose in Tris-acetate-EDTA buffer and visualized with ethidium bromide staining. Amplicons were extracted from 1% agarose gels and purified using the AxyPrep DNA Gel Extraction Kit (Axygen Biosciences, CA, USA) according to the manufacturer’s instructions and quantified using QuantiFluor-ST (Promega, Madison, WI, USA). Purified amplicons were then paired-end sequenced (2 × 300) on an Illumina MiSeq platform according to standard protocols.

### 2.4. Bioinformatics Analysis of the Sequence Data

In the present study, raw FASTQ files were demultiplexed and quality-filtered with the following criteria: (i) The 300-bp reads were truncated at any site that had an average quality score < 20 over a 50-bp sliding window, and truncated reads < 50 bp were discarded; (ii) exact barcode matching was required, and any 2-nucleotide mismatch in primer matching and reads containing ambiguous characters were removed; and (iii) only sequences that overlapped by more than 10 bp were assembled according to their overlap sequences. The length of over 89% of the total valid sequence was between 421 and 440 bp, while around 10% of sequences were ranged between 441 and 460 bp. Operational taxonomic units (OTUs) were clustered with 97% similarity cutoff using Uparse algorithm (version 7.1, http://drive5.com/uparse/), and chimeric sequences were identified and removed using UCHIME. The taxonomy of each *mcr*A gene sequence was analyzed by the RDP Classifier against the FunGene Database using a confidence threshold of 70% [13]. Good’s coverage and rarefaction curves were determined to estimate the coverage and sampling effort. QIIME (version 1.17) was also used to calculate the archaeal population diversity (Simpson’s diversity index), evenness (Shannon’s diversity index), and richness (Chao1 and Ace index). Venn diagram was constructed according to Oliveros [14] to show the shared and unique OTUs among samples. Heatmap analysis and identification of significant features were also used to determine changes among samples. The data obtained in the present study have been submitted to NCBI (submission ID: SUB 3898364, BioProject ID: PRJNA449795).

### 2.5. Statistical Analyses

Data were analyzed using SPSS software (version 22.0 for Windows, SPSS Inc., Chicago, IL, USA). Dietary DM intake, milk yield, ECM and milk composition were analyzed the using one-way ANOVA package. The model used was as follows:

Y*_ij_* = μ + A*_i_* + T*_j_* + e*_ij_* (2)

where *Y_i_* = the observations for the dependent variable, μ = overall mean, A*_i_* = the random animal effect, T*_j_* = the fixed effect of the *j*th *Moringa oleifera* amount (treatment, *j* = 3, 6, or 9% *w/w*), and *e_ij_* = the random residual assumed to be normally distributed with mean zero.

Relative abundance data of fecal methanogenic archaea are presented as percentages/proportions. These data were analyzed using Welch’s t-test package, and the model was as follows:

Y*_ij_* = μ + S*_i_* + T*_j_* + e*_ij_* (3)

where *Y_i_* = the observations for the dependent variable, μ = overall mean, S*_i_* = the random effect of sampling, T*_j_* = the fixed effect of treatment *j*, and *e_ij_* = the random residual assumed to be normally distributed with mean zero.

Treatment means were considered statistically different at *p* < 0.05, and SEM values are presented with *p* values.

## 3. Results

### 3.1. Feed Intake and Milk Yield and Composition 

The DM intake, milk yield, and ECM were similar among the four treatments (Table 2). For the milk composition, fat content (38.2 g/kg) was highest in the M6 treatment (*p* < 0.05) compared with the other three treatments. Protein, lactose, and total solid content did not differ significantly among the 4 treatments.

### 3.2. Composition and Dynamics of Fecal Methanogen Community

A total of 450,500 high-quality sequences with an average length of 439.5 bp were obtained. The Good’s coverage indices obtained from each treatment were all above 0.999, indicating a high-quality of sampling and sequencing. The richness and diversity indices obtained for fecal samples of cows fed different levels of *M. oleifera* are presented in Table 3. Richness indices after the 4 treatments did not differ significantly, although the control group had relatively higher values of Ace and Chao (21.05 and 20.63). By contrast, Shannon’s index was highest for the M9 group (1.783; *p* < 0.05) and similar among the control, M3, and M6 groups (1.653, 1.438, and 1.628, respectively). The Simpson index was lowest for the M9 group (*p* < 0.05) and did not differ significantly among the control, M3, and M6 groups.

**Figure 1:**
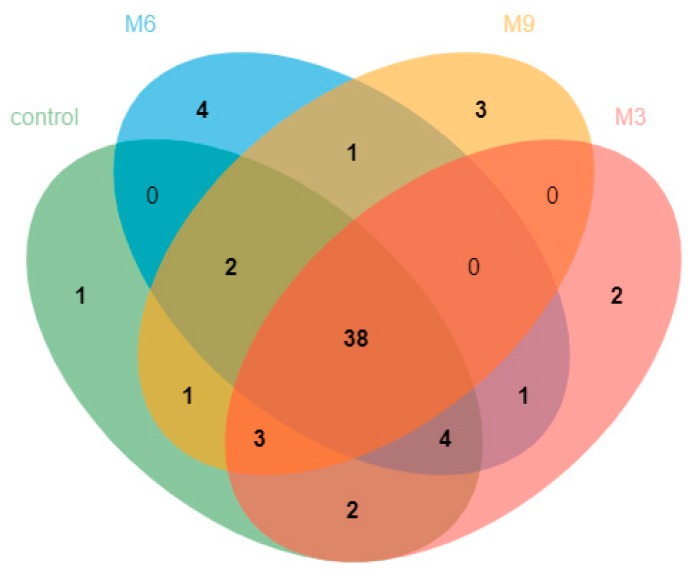
Venn diagram representation of the shared and exclusive Operational taxonomic units (OTUs) at 97% similarity level of four groups: Control (without *Moringa oleifera* supplementation), M3 (a low dose of *Moringa oleifera* supplementation, 3% *w/w*), M6 (a medium dose of *Moringa oleifera* supplementation, 6% *w/w*), M9 (a high dose of *Moringa oleifera* supplementation, 9% *w/w*) in lactating Holstein dairy cows.

**Figure 2:**
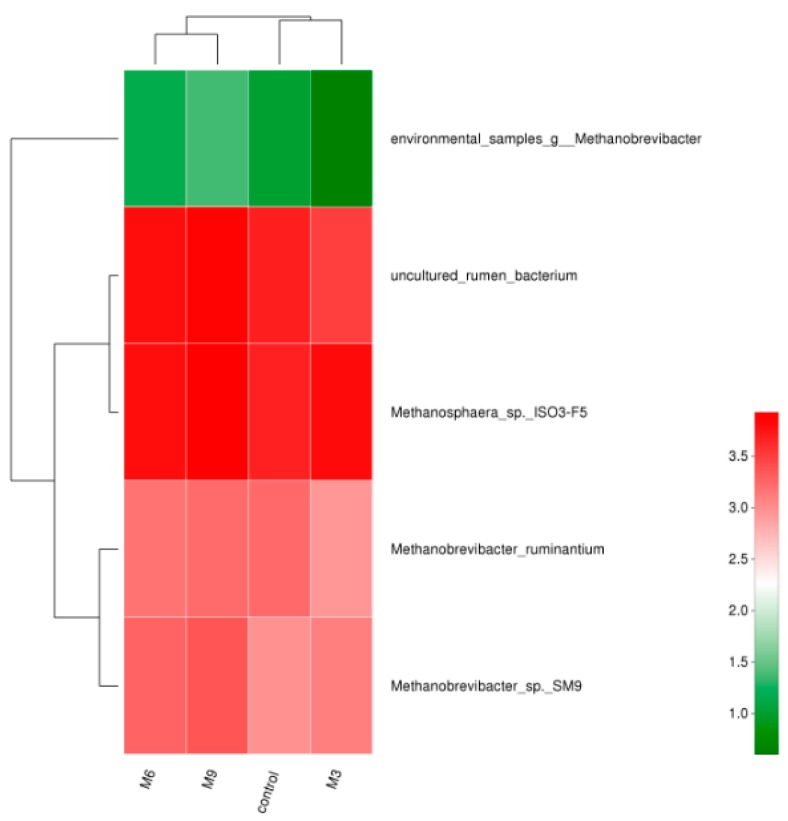
Heatmap under the species level of the four treatments of the lactating Holstein dairy cows fecal sample (control: without *Moringa oleifera* supplementation; M3: a low dose of *Moringa oleifera* supplementation, 3% *w/w*; M6: a medium dose of *Moringa oleifera* supplementation, 6% *w/w*; M9: a high dose of *Moringa oleifera* supplementation, 9% *w/w*).

A Venn diagram constructed using the OTUs for the sequences from the fecal samples is presented in Figure 1. Shared and unique OTUs were represented at a 97% similarity level among the 4 treatments. A total of 51, 50, 48, and 50 OTUs were found for the control, M3, M6, and M9 treatments, respectively, and 38 OTUs were common to all 4 treatments. In pairwise comparisons of treatments, 43 OTUs were shared between the M3 and M6 treatments, 41 between the M6 and M9 treatments, and 41 between the M3 and M9 treatments. Between the control and treatment M3, M6, or M9, respectively, 47, 44, and 44 OTUs were shared. In addition, a heatmap was constructed to determine the relationship between OTUs and experimental treatments based on the log-transformed sequence abundance and is presented based on the species level at the 97% similarity level (Figure 2). The heatmap showed a change in the abundance of *Methanosphaera* sp. ISO3-F5, *Methanobrevibacter ruminantium*, and *Methanobrevibacter* SM9, indicating that supplementation of *M. oleifera* in the diet resulted in archaeal populations distinct from the control, the same as shown in the abundance analysis.

The relative abundance of fecal methanogenic archaea in the dairy cows with different supplements of *M. oleifera* based on the *mcr*A gene sequencing is listed by taxonomic level in Table 4; three phyla, four orders, four families, five genera, and seven species were determined. The predominant archaeal family across the four treatments in the order *Methanobacteriales* was *Methanobacteriaceae*, with species *Methanosphaera* sp. ISO3-F5, *Methanobrevibacter* sp. SM9, and *Methanobrevibacter ruminantium*. 

Specifically, at the order level, no significant difference was observed among the four treatments in terms of *Methanobacteriales* and *Methanomicrobiales*. However, *Methanobacteriales* was relatively more abundant in the M3 treatment (0.677) when compared with the control (0.582), M6 (0.615), and M9 (0.606) treatments. At the genus level, the *Methanocorpusculum* was the third most abundant genus with an average value of 15.0%, 6.5%, 14.2%, and 9.9% for the control, M3, M6, and M9 treatments, respectively. Similar values were obtained for *Methanobrevibacter* among the 4 treatments, whereas the abundance of *Methanosphaera* was significantly higher in the M3 and M9 treatments (0.297 and 0.293, respectively, *p* < 0.05). There was no significant difference between the control and M6 group or between M3 and M9 groups. The species abundance of *Methanosphaera* sp. ISO3-F5 was significantly higher in the M3 and M9 groups (0.297 and 0.291, respectively) than in the control and M6 groups (0.191 and 0.215, respectively, *p* < 0.05), but no significant difference was found between the control and M6 groups or between the M3 and M9 groups. The abundance of *Methanobrevibacter ruminantium* was significantly higher in the control than in the other three groups (*p* < 0.05), but there was no difference among the M3, M6 and M9 group. In addition, values for *Methanobrevibacter SM9* were similar across the four groups but accounted for less than 10% of the total species in each group.

## 4. Discussion

### 4.1. Feed Intake, Milk Yield and Composition

Application of agricultural by-products in the ruminant production system has been extensively investigated because of their relatively high biomass yield and low cost. In this research, the production, and fecal methanogenic archaea were examined when lactating dairy cows were subjected to different levels of supplementation of *M. oleifera* in the diet. The milk fat content in the M6 treatment was significantly higher than that in the control and M3 treatments, although M6 treatment had relatively higher values of milk yield and ECM. Several consistent results were reported previously that supplementation of *M. oleifera* enhanced milk yield and milk composition. For example, Cohen-Zinder et al. [15] found a significant increase in milk yield, milk fat and protein content in lactating cows offered an *M. oleifera* diet. Azzaz et al. [3] reported that milk and total solid yield increased by 11.3 and 17.7% in lactating ewes fed a supplement of 15 g/kg DM *M. oleifera*. This positive effect of *M. oleifera* on production performance can be attributed to improved feed intake, apparent nutrient digestibility, and ruminal fermentation conditions [4,15]. Moderate concentrations of phenolics and tannins in *M. oleifera* exhibited antioxidant and antimicrobial properties, which have beneficial effects in productive ruminants [16]. Aerts et al. [17] reported that ruminal methanogenesis would be inhibited by phenolics and tannins, which leading to repartition of consumed energy in CH_4_ and milk production. This was in agreement with the results of Shaani et al. [7] that improved fat yield and ECM production efficiency resulted from inhibition of CH_4_ production by ruminal methanogenic bacteria.

### 4.2. Fecal Methanogenic Composition and Dynamics 

The composition and function of ruminal methanogens have been studied in great detail, whereas less is known in the lower gastrointestinal tract (GIT) of ruminants [18]. Previous research showed relationships between methanogens in feces and those present in the pregastric compartments [19,20]. In the present study, the functional *mcr*A gene sequencing technique was used, and the results revealed the presence of *Methanobrevibacter*, *Methanosphaera*, and *Methanocorpusculum* in the feces of lactating dairy cows. These results were consistent with previous studies that detected two phyla and six genera in the feces of multiparous dairy cows, whereas fecal *mcr*A sequences had the closest similarity to *Methanocorpusculum*, *Methanobacterium*, and *Methanobrevibacter* species [9]. Jin et al. [9] found that fecal archaeal community was predominated by *Methanobrevibacter* (86.9% of the total sequence reads) and *Methanocorpusculum* (10.4%). A bTEFAP pyrosequencing study reported the dominant methanogens of *Methanobrevibacter*, *Methanophaera*, and *Methanobacteriaceae* in the hindgut of goats [21]. In addition, Mohammadzadeh et al. [10] suggested that the fermentation characteristics as digesta pass from the rumen into the small intestine and out of the animal would affect methanogen diversity. The sequencing technique may also influence the results, although the *mcr*A gene-based approach was thought to be comparable to the 16S rRNA gene for phylogenetic studies.

Different from previous studies, however, the present study demonstrated that *Methanobrevibacter* sequence made up approximately 35% of the total. Guzman et al. [22] hardly detected *Methanobrevibacter* in the feces of calves in the first 3 days after birth. In mature cows, *Methanobrevibacter* represented 62% of the rumen archaea, and they were among the most important and dominant archaea in the rumen fluid. A higher percentage was also found in the hindgut of goats with *Methanobrevibacter* accounting for 74.8% of the total sequenced reads, while Jin et al. [9] reported that *Methanobrevibacter* was the dominant phylotype at the genus level accounting for over 86% of the total sequence reads. These results indicated that the presence and abundance of *Methanobrevibacter* may be influenced by dietary composition, enteric fermentation, and even environmental factors.

At the genus level, the relative abundance of *Methanosphaera* and *Methanocorpusculum* was generally around 20% and 15% in the fecal sample of dairy cows, higher than previously reported from the rumen and fecal samples of ruminants [19]. For example, Liu et al. [19] did not detect *Methanocorpusculum* in the rumen but made up only 2% of the archaeal community in the feces of sheep. Jin et al. [9] reported a very low content of *Methanosphaera* in the feces of lactating cows (0.8%). The great diversity of the fecal microbiota can be attributed to various factors such as animal breeds, diet sources, and composition.

### 4.3. Effect of Moringa Oleifera Supplementation on Fecal Methanogenic Archaea 

A range of studies reported that the fecal microbial relative abundance and composition were affected by types of diet or different dietary supplementations [23,24,25]. For example, the fecal microbial community structure was significantly changed as cattle were fed either high-grain diets or high-forage diets [24]. In our *mcr*A gene-based sequencing study of the methanogenic archaeal community in the feces of lactating cows, the richness indexes remained similar when *M. oleifera* was added at different levels to the diet. However, the Simpson diversity index was significantly lower compared to the control treatment. Changes in the fecal methanogen diversity might be dependent on nutrient contents and the fermentation profile of the fecal samples as a result of secondary metabolites from *M. oleifera*, similar to the alteration of the ruminal environment and microbial activity when *M. oleifera* was fed to ruminants [1,9]. For example, previous results showed that feeding *M. oleifera* plant improved nutrient digestibility and increased SCFA concentration in the rumen of goat, which resulted in the growth of propionate-producing bacterial species and inhibition of CH_4_-producing archaea [26]. The high protein (241–277 g/kg DM) and polyphenol content make *M. oleifera* a high-quality feed resource [27]. Bioactive products such as saponins (80 g/kg) and tannins (12 g/kg) in *M. oleifera* leaves have an antimicrobial function and play a key role in improving nutrient digestibility and fermentation efficiency [28,29]. When steers were supplemented with up to 30 g/day tea saponin, daily CH_4_ emission (g/day) was reduced by 18%, and yield (CH_4_/DM intake, g/kg) was reduced by 22% [30]. In the present study, as demonstrated in our previous experiment, the calculated saponin intake was 144 g/day when 9% of *M. oleifera* was included in the diet. We thus assumed that the composition and distribution of methanogenic archaea changed along the gastrointestinal tract, and methanogenesis and CH_4_ emissions would be inhibited by such a large amount of saponin intake. However, feeding saponin-containing *Yucca schidigera* and *Quillaja saponaria* powder (10 g/kg DM) differed little in CH_4_ emission (g/day) and yield (CH_4_-E/GE intake) from the basal diet [31]. This discrepancy of CH_4_ reduction may be attributed to the source and the actual saponin content. Further research is needed to compare responses when similar saponin sources or supplementation levels are used.

When *M. oleifera* was added to the diet, *Methanosphaera* and *Methanosphaera* sp. ISO3-F5 increased in abundance but *Methanobrevibacter ruminantium* decreased compared with the control group. *Methanobrevibacter ruminantium* is a strict anaerobe that can produce CH_4_ from H_2_, CO_2_, and formate and has a close syntrophic association with protozoa [32]. Secondary metabolites from *M. oleifera* such as saponins and tannins have antiprotozoal properties that affect cell membrane integrity [33]. Soliva et al. [34] found that approximately 30% of ciliate protozoa concentration was reduced when extracted *M. oleifera* was added in vitro experiment. This result was in accordance with the inhibitory effect of saponin on ruminal ciliate protozoa population in cattle or sheep [35]. In line with a range of in vivo and in vitro experiments adding different sources and levels of secondary metabolites from *M. oleifera*, *Methanosphaera* sp. ISO3-F5 increased as levels of *M. oleifera* increased. As one of the main methylotrophic methanogens, *Methanosphaera* sp. ISO3-F5 was found to be associated with different bacteria including members of *Lachnospiraceae* [36]. Thus, it would be interesting to examine the pectin content of *M. oleifera* to see its influence on *Methanosphaera* sp. ISO3-F5 abundance. In addition, more future work will be needed to investigate the interaction between some specific methanogens and ruminal fermentation conditions, which may help for a better understanding of rumen microbial metabolic function and development of CH_4_ mitigation approaches.

## 5. Conclusions

This study demonstrated that different supplementation levels of *Moringa oleifera* in the diet achieved similar feed intake, milk production, but adding 6% of *Moringa oleifera* improved milk fat content. The fecal methanogenic archaea diversity changed as the increased abundance of the *Methanosphaera* genus and *Methanosphaera* sp. ISO3-F5 species was induced by secondary metabolites of *Moringa oleifera* in the diet. This study provided some basic information on the utilization of *Moringa oleifera* as forage resources for dairy cows, and helped to elucidate the interaction between methanogens and other microbes, regulation of microbial metabolic function and methane emissions.

## Figures and Tables

**Table 1:** Ingredients and nutrient composition of the experimental diets.

Items	Dietary *Moringa Oleifera* Content			
	0	3%	6%	9%
Ingredients, % of DM				
Ground corn	21.2	21.7	22.1	22.7
Soybean meal	10.5	11.6	12.7	13.8
DDGS	8.4	8.4	8.4	8.4
Cottonseed meal	7.5	7.5	7.5	7.5
Palm fat	7.5	7.5	7.5	7.5
Beet pulp	4.7	4.7	4.7	4.7
Alfalfa hay	20.5	17.1	13.7	10.2
Corn silage	16.8	15.7	14.5	13.4
*Moringa oleifera*	0.0	3.0	6.0	9.0
Premix ^1^	2.0	2.0	2.0	2.0
Sodium hydrogen carbonate	0.6	0.6	0.6	0.6
Calcium hydrogen phosphate	0.2	0.2	0.2	0.2
Sodium chloride	0.3	0.3	0.3	0.3
Nutrient composition ^2^				
CP	178.70	179.00	179.30	179.60
EE	44.80	46.90	48.80	50.90
Ash	76.80	84.60	73.50	79.20
NDF	437.00	434.30	431.70	428.60
ADF	211.30	214.30	217.20	219.30
NE_L_, MJ/kg	6.60	6.61	6.62	6.63

^1^ One kilogram of premix contained the following: 100,000 IU VA; 40,000 IU VD; 1000 IU VE; 330 mg Fe; 250 mg Cu; 400 mg Mn; 500 mg Zn; 10 mg Se; 10 mg I; 5 mg Co; ^2^ All values were measured from the monthly total mixed ration (TMR) samples, while NE_L_ (net energy for lactation) was calculated based on Ministry of Agriculture (MOA) of P.R. China individual feedstuffs recommendations based on chemical composition (MOA, 2004).

**Table 2:** Effects of supplementation of *Moringa oleifera* rachises and twigs on the dry matter intake, milk yield and composition of Holstein dairy cows.

Item ^1^	Treatment ^2^				SEM ^3^	*p*-Value
	Control	M3	M6	M9		
DM intake, kg/day	20.6	20.9	20.7	19.3	0.53	0.09
Milk yield, kg/day	29.6	29.7	31.0	28.6	0.46	0.46
ECM kg/day	31.7	32.2	34.5	31.4	0.57	0.69
Milk composition, %						
Fat	3.54 ^b^	3.62 ^b^	3.82 ^a^	3.68 ^ab^	0.07	0.04
Protein	3.65	3.70	3.66	3.70	0.03	0.49
Lactose	5.10	5.08	5.08	5.05	0.03	0.14
Total solid	13.0	13.1	13.2	13.1	0.08	0.13

^1^ DM = dry matter; ^2^ M3, M6, and M9 = dietary *Moringa oleifera* supplementation of 3%, 6%, and 9% *w/w*; Means within rows lacking common superscript differ (*p* < 0.05); ^3^ SEM = standard error of means.

**Table 3:** Effects of supplementation of *Moringa oleifera* rachises and twigs on the diversity indices of fecal methanogenic archaea based on *mcr*A gene sequences of lactating Holstein dairy cows.

Item ^1^	Treatment ^2^				SEM ^3^	*p*-Value
	Control	M3	M6	M9		
Ace	21.05	19.78	15.30	14.35	1.788	0.510
Chao	20.63	19.25	19.75	18.25	0.569	0.561
Shannon	1.653 ^b^	1.438 ^b^	1.628 ^b^	1.783 ^a^	0.0570	0.019
Simpson	0.310 ^a^	0.393 ^a^	0.328 ^a^	0.265 ^b^	0.0199	0.014

^1^ Indices of Ace, Chao, Shannon, and Simpson were calculated to measure alpha diversity of the methanogens in the sample; ^2^ M3, M6, and M9 = dietary *Moringa oleifera* supplementation of 3%, 6%, and 9% *w/w*; Means within rows lacking common superscript differ (*p* < 0.05); ^3^ SEM = standard error of means.

**Table 4:** Effects of supplementation of *Moringa oleifera* rachises and twigs on the relative abundance of fecal methanogenic archaea based on *mcr*A gene sequences of lactating Holstein dairy cows ^1^.

Item	Treatment				SEM ^2^	*p*-Value
	Control	M3	M6	M9		
Phylum						
Euryarchaeota	0.734	0.743	0.757	0.705	0.022	0.883
Uncultured rumen archaea	0.216	0.150	0.213	0.272	0.016	0.456
Unclassified	0.051ab	0.108a	0.03ab	0.023b	0.015	0.172
Order						
Methanobacteriales	0.582	0.677	0.615	0.606	0.023	0.561
Methanomicrobiales	0.150	0.065	0.142	0.099	0.026	0.676
Uncultured rumen archaea	0.216	0.150	0.213	0.272	0.016	0.456
Family						
Methanobacteriaceae	0.582	0.677	0.615	0.606	0.023	0.561
Methanocorpusculaceae	0.150	0.065	0.142	0.099	0.026	0.676
Uncultured rumen archaea	0.216	0.150	0.213	0.272	0.016	0.456
Genus						
*Methanobrevibacter*	0.391	0.380	0.400	0.315	0.026	0.698
*Methanosphaera*	0.191b	0.297a	0.215b	0.291a	0.016	0.016
*Methanocorpusculum*	0.150	0.065	0.142	0.099	0.026	0.676
Uncultured rumen archaea	0.216	0.150	0.213	0.272	0.016	0.456
Species						
Unclassified *Methanobrevibacter*	0.274	0.279	0.281	0.172	0.031	0.569
*Methanosphaera* sp. ISO3-F5	0.191b	0.297a	0.215b	0.291a	0.016	0.016
Unclassified *Methanocorpusculum*	0.150	0.065	0.142	0.099	0.026	0.676
Uncultured rumen archaea	0.216	0.150	0.213	0.272	0.026	0.456
*Methanobrevibacter* sp. SM9	0.038	0.060	0.065	0.080	0.007	0.258
*Methanobrevibacter ruminantium*	0.078a	0.041b	0.054b	0.062b	0.014	0.035
Unclassified	0.051	0.108	0.030	0.023	0.015	0.172

^1^ M3, M6, and M9 = dietary *Moringa oleifera* supplementation of 3%, 6%, and 9% *w/w*; ^2^ SEM = standard error of means.
